# The use of nanoscale fluorescence microscopic to decipher cell wall modifications during fungal penetration

**DOI:** 10.3389/fpls.2014.00270

**Published:** 2014-06-18

**Authors:** Dorothea Ellinger, Christian A. Voigt

**Affiliations:** Phytopathology and Biochemistry, Biocenter Klein Flottbek, University of HamburgHamburg, Germany

**Keywords:** callose, cell wall integrity, FRET, innate immunity, localization microscopy, powdery mildew, plant defense, super-resolution

## Abstract

Plant diseases are one of the most studied subjects in the field of plant science due to their impact on crop yield and food security. Our increased understanding of plant–pathogen interactions was mainly driven by the development of new techniques that facilitated analyses on a subcellular and molecular level. The development of labeling technologies, which allowed the visualization and localization of cellular structures and proteins in live cell imaging, promoted the use of fluorescence and laser-scanning microscopy in the field of plant–pathogen interactions. Recent advances in new microscopic technologies opened their application in plant science and in the investigation of plant diseases. In this regard, *in planta* Förster/Fluorescence resonance energy transfer has demonstrated to facilitate the measurement of protein–protein interactions within the living tissue, supporting the analysis of regulatory pathways involved in plant immunity and putative host–pathogen interactions on a nanoscale level. Localization microscopy, an emerging, non-invasive microscopic technology, will allow investigations with a nanoscale resolution leading to new possibilities in the understanding of molecular processes.

## INTRODUCTION

The plant cell wall and its outer cuticle represent the first line of defense to biotic and abiotic stress. Based on its crucial role in plant defense, the cell wall also constitutes a primary target of plant pathogen attack and is constantly subject to various extrinsic, physical forces. While pathogens try to enter the cell to establish infection structures for further colonization of the tissue, which can be associated with a reprogramming of the plant metabolism for its own purpose, as in the case of obligate biotrophic and hemibiotrophic pathogens with their release of effectors (Giraldo and Valent, 2013), the plant responds to the attack by a variety of cell wall-associated defense reactions.

Even though we already have a detailed insight into the processes and signaling pathways that follow recognition of pathogens by plasma membrane-bound receptors (Hamann, 2012; Wolf et al., 2012), we only have little knowledge about processes that occur directly at the cell wall. In recent years, growing evidence suggests that a mechanism for cell wall integrity may exists, which monitors and maintains functional integrity of the cell and includes restructuring and rebuilding of cell wall components (Knepper and Day, 2010; Steinbrenner et al., 2012). In this regard, the plant cell wall seems to be more dynamic as previously expected. In response to pathogen attack, the main cell wall polymer cellulose, a (1,4)-β-glucan, forms a three-dimensional network with the (1,3)-β-glucan callose (Eggert et al., 2014), a cell wall polymer that is directly associated with the plant’s innate immunity (Hardham et al., 2007). A prerequisite of these advances in cell wall visualization is the increasing number of advanced molecular dyes and techniques that have become available for high resolution imaging of cell wall integrity processes and for localization of individual cell wall components.

Here, we describe the role that Förster/Fluorescence resonance energy transfer (FRET) and localization microscopy has played and probably is going to play in investigating plant–pathogen interaction by highlighting processes occurring at the plant cell wall and being part of cell wall integrity mechanisms. In contrast to scanning electron microscopy, transmission electron microscopy, or atomic force microscopy, FRET and localization microscopy are both suitable to visualize processes and interactions between different components with a resolution in the submicron to nanometer scale in live cell imaging where the tissue is still intact and in its native state maintaining the full functionality of enzymes as well as arrangement of cell wall fibrils and other components.

This perspective focuses on the new and emerging possibilities in subcellular investigation of plant–pathogen interactions and the understanding of how individual molecules, such as callose and cellulose, and its regulating enzymes allow plants to perceive pathogens and pathogens to infect their hosts.

## FRET MICROSCOPY

Förster/Fluorescence resonance energy transfer is commonly used to image the interaction of fluorescent labeled molecules or proteins in living cells. The physical principle of FRET is a distance-dependent interaction between the electronic excited states of two dye molecules where excitation energy is transferred from a donor molecule to an acceptor molecule without emission of a photon. This transfer of energy only happens if (i) the absorption spectrum of the acceptor overlap with the fluorescence emission spectrum of the donor and (ii) donor and acceptor molecules are in close proximity (1–10 nm; Jares-Erijman and Jovin, 2003; Grecco and Verveer, 2011). Donor and acceptor molecules might be either fused to different putative interaction partners or linked with each other by a spacer. In the first case, energy transfer takes place as soon as both partners bind each other. In second case, changes in protein folding induced by shifts in the redox state, tension, or pH reduce the distance of the FRET pair partners to a level that allows energy transfer (Gjetting et al., 2012). The FRET pair can function as a FRET sensor that generally consists of a substrate-specific binding domain, which is flanked by a suitable donor on the one and acceptor on the other site. Substrate binding causes a change in distance or orientation of the two fluorophore that is translated into a measurable change in energy transfer. FRET sensors have been developed for a large spectrum of substrates and are used to analyze dynamic processes in mammals and plant cells (Okumoto et al., 2012; Jones et al., 2013; Okumoto, 2014). Interestingly, mammal sensors were successfully used in plant cells to identify new sugar transporters (Chen et al., 2010), highlighting the comparability of basic cellular mechanisms in different biological kingdoms.

### FRET IN ANALYZING PROTEIN–PROTEIN INTERACTION OF PLANT PENETRATION RESISTANCE

Over 150 years ago, deBary (1863) discovered cell wall thickenings in plants, so called papillae, at sites where fungal pathogens penetrated through the cell wall. Chemical analyses of papillae have identified callose, a (1,3)-β-glucan with some (1,6)-branches (Aspinall and Kessler, 1957), as the most common constituent among others, which may also include protein (e.g., peroxidases, antimicrobial thionins), phenolics, and other constituents (Aist and Williams, 1971; Mercer et al., 1974; Aist, 1976; Mims et al., 2000). The formation as well as degradation of papilla requires a high spatial and temporal regulation of transport processes between the infection site, the plasma membrane, and the trans-Golgi network. Alteration or disruption of these regulatory processes cannot only result in increased susceptibility to pathogen attack, but also induce complete penetration resistance to powdery mildews, which are biotrophic fungal pathogens. This was illustrated in studies with *Arabidopsis* (*Arabidopsis thaliana*) where overexpression of *GSL5* (*GLUCAN SYNTHASE-LIKE 5*, also known as *POWDERY MILDEW RESISTENT 4*), a gene encoding a stress-induced callose synthase (Jacobs et al., 2003; Nishimura et al., 2003), resulted in early and elevated callose biosynthesis at sites of attempted penetration by the adapted powdery mildew *Golovinomyces cichoracearum* and the non-adapted powdery mildew *Blumeria graminis* f.sp. *hordei*. These enhanced callose deposits prevented pathogen ingress (Ellinger et al., 2013). Penetration resistance to fungal pathogens was also observed after disruption of mildew resistance locus O (MLO) protein family members in *Arabidopsis* infected with *G. cichoracearum* and *G. orontii* as well as the necrotrophic fungus *Botrytis cinerea* (Consonni et al., 2006, 2010), in tomato (*Solanum lycopersicum*) after infection with the powdery mildew *Oidium neolycopersici* (Bai et al., 2008), and in barley (*Hordeum vulgare*) after *B. graminis* f.sp. *hordei* infection (Jørgensen, 1992; Piffanelli et al., 2004). MLO proteins have been characterized as a family of plasma membrane-localized MLO proteins that are required for successful entry of adapted powdery mildew species in leaf epidermal cells (Panstruga, 2005).

**FIGURE 1:**
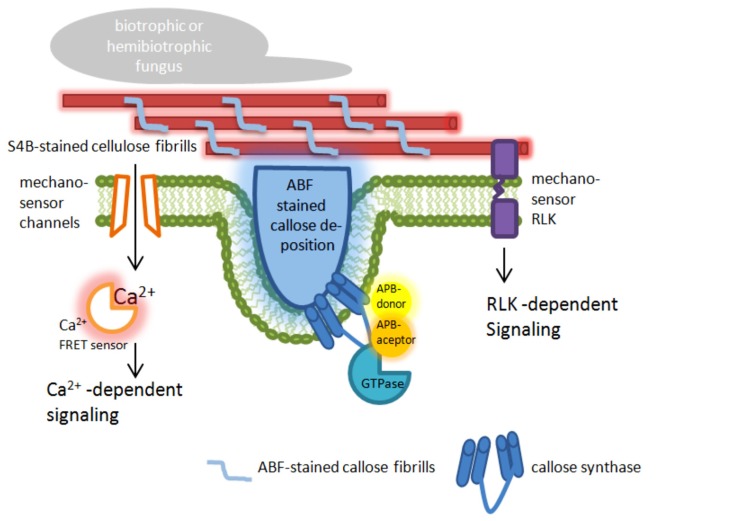
**Targets for nanoscale microcopy tools to analyze plant immunity-related cell wall modifications.** The presented model highlights possible targets at the plasma membrane and the cell wall in epidermal leaf cells of plants attacked by pathogens. ABF, aniline blue fluorochrome [fluorescent dye specific for the (1,3)-β-glucan callose]; S4B, pontamine fast scarlet 4B [fluorescent dye specific for the (1,4)-β-glucan cellulose]; APB, acceptor photo bleaching; FRET, Förster/Fluorescence resonance energy transfer; RLK, receptor-like kinase.

Förster/Fluorescence resonance energy transfer microscopy has been used to analyze the recruitment and interaction dynamics of components that contribute to plant penetration resistance, which strongly promoted this technique in the field of plant–pathogen interaction. Using FRET-acceptor photo bleaching (APB; Karpova et al., 2003) and FRET-Fluorescence lifetime imaging microscopy (FLIM; Becker, 2012), new spatiotemporal information about the interaction of MLO and calmodulin, a cytoplasmatic calcium sensor (Cheval et al., 2013), and a new function of MLO was obtained (Iwai and Uyeda, 2008), which had remained undetected before using these advanced microscopic technologies. A prominent field of FRET-APB application is to verify dynamic protein–protein interaction between cytosolic and membrane-bound proteins, like the interaction of the endoplasmic reticulum (ER)-resident BAX INHIBITOR-1 (BI-1) protein with the cytochrome oxidase CYP83A1 during inoculation with the adapted powdery mildew fungus *Erysiphe cruciferarum* (Weis et al., 2013). A further field of FRET-APB application could be the localization of membrane-bound enzymes or enzyme complexes that are involved in reorganization and reinforcement of the cell wall after pathogen attack. In this regard, the stress-induced callose synthase GSL5 from *Arabidopsis* could be a suitable target. Its involvement in pathogen-induced cell wall rearrangements was clearly shown (Jacobs et al., 2003; Nishimura et al., 2003; Eggert et al., 2014); and a successful fluorescence-tagging was also demonstrated (Ellinger et al., 2013; Naumann et al., 2013). Because quantitative proteomics of plasma membrane microdomains from poplar (*Populus trichocarpa*) cell suspension cultures suggested a localization of callose synthases in lipid rafts (Srivastava et al., 2013), a FRET analysis of tagged GSL5 and lipid raft-resident protein used as markers could reveal whether this specific localization would also occur in intact plant tissue. In addition, this analysis might provide information about the mechanisms of enzyme translocation that was already observed during infection (Ellinger et al., 2013). Because it has been suggested that a callose synthase complex is formed at sites of callose biosynthesis (Verma and Hong, 2001), FRET-ABP can be used as a microscopic tool to screen for possible interaction partners of the callose synthase GSL5 at sites of attempted fungal penetration. A promising target for a screening would be monomeric GTPases (Figure 1) that were already identified as putative interaction and complex forming partners in *Saccharomyces cerevisiae* where a GTPase from the Rho family might control the phosphorylation status of the callose synthase (Calonge et al., 2003), and in *Arabidopsis* where the Rho-like GTPase Rop1 might be involved in regulating callose biosynthesis of GSL6 at cell plate through interaction with the UDP-glucose transferase UGT1 (Hong et al., 2001).

### FRET FOR SENSING ION INFLUX AND MECHANICAL STRESS

During entry of pathogenic fungi into plant tissue, a localized deformation of the cell surface occurs. The perception of those punctual mechanical signals induces a dynamic protein interaction (as described above) and a very rapid reorganization of actin microfilaments, ER, and peroxisoms (Koh et al., 2005). This is also associated with ion flux within the plant cell (Meng and Sachs, 2011). Using the FRET based Ca^2^^+^ sensor Yellow Cameleon 3.6 (Nagai et al., 2004), it was demonstrated in root hairs that localized cell wall deformation induced a monophasic Ca^2^^+^ increase starting from the site of stress and spreading through the cytoplasm, which finally activated extracellular production of reactive oxygen species at the cell wall (Monshausen et al., 2008, 2009). At the site of fungal ingress, also callose is deposited in the apoplastic space (Figure 1). The amount of deposited callose depended on the cytoplasmatic Ca^2^^+^ level because the presence of chelators or inhibitors of Ca^2^^+^ channels reduced callose biosynthesis (Mercer et al., 1974; Mims et al., 2000). It has been speculated that possible stress-activated Ca^2^^+^-permeable channels might be gated by changes in membrane tension (Bhat et al., 2005).

The sensing of mechanical stress and the resulting induction of callose biosynthesis or cellulose remodeling likely occurs via cell wall sensors. These mechanosensory proteins with their putative carbohydrate-binding domains, like lectin receptor kinases (Vaid et al., 2013), cell wall-associated kinases (Kohorn and Kohorn, 2012), or the THESEUS 1 receptor-like kinase (Cheung and Wu, 2011), might be linked to the cytoskeleton or to glycosylated proteins and polysaccharides of the cell wall. Here, they could transmit information about deformation of the cell wall via kinase-dependent phosphorylation of target proteins. Although neither phosphorylation of cell wall integrity target proteins nor tension-depended activation of cell wall sensors was shown so far in plant–fungus interactions, functional FRET sensors for phosphorylation (Sato and Umezawa, 2004; Mirabet et al., 2011) as well as tension (Nagai et al., 2004; Monshausen et al., 2009; Ellinger et al., 2013; Monshausen and Haswell, 2013) already exist. As stated by Ehrhardt and Frommer (2012), these tools have to make useable to the plant science community.

However, a major challenge in investigating cell wall integrity during plant–pathogen interaction using FRET technology is the autofluorescence in photosynthetic active tissue and especially of cell wall material. Therefore, fluorophores for these applications have to be carefully selected. An alternative would be the use of bioluminescent proteins, like the Ca^2^^+^-sensitive aequorin (Kunkel et al., 2007) where excitation is caused by a chemical reaction instead of light. In general, it has to be considered that FRET efficiency strongly depends on the distance that separates the FRET pair and the spatial orientation of the fluorophores. In most applications that we referred to in this article, fluorophores were fused to the proteins of interest. However, this protein modification may change its conformation, activity, or even stoichiometry in a protein-protein-interaction. Therefore, a lack of FRET efficiency cannot only indicate a non-interaction of proteins but also an inappropriate protein modification. As a consequence, various constructs with alternative fusion sites or linker usage would have to be tested to eventually distinguish between inappropriate modification and actual protein non-interaction. Nevertheless, for both, negative and positive FRET signals, appropriate controls have to be included; and results of FRET experiments should be verified by other methods like protein co-precipitation experiments in case of protein-protein interactions.

## LOCALIZATION MICROSCOPY

A great advantage of FRET microscopy is the possibility to resolve molecule or protein interaction at nanoscale in live cell imaging. However, the resolution of the imaging system itself is not increased in FRET application. As a consequence, it is possible to determine that a specific interaction between the partners of FRET pair occurs in the cell, but it is not possible to determine on a nanoscale level where exactly the interaction takes place in the cell.

To overcome this limitation, new imaging technologies are now available, which allow the localization of molecules and proteins below the diffraction limit and are commonly referred to as super-resolution microscopy (Hell, 2007; Agrawal et al., 2013; Requejo-Isidro, 2013). These new microscopic techniques include stimulated emission depletion fluorescence (STED) microscopy with a possible resolution of 35 nm in the far field (Hell and Wichmann, 1994), but without reports of successful application on intact plant tissue due to the relatively high laser energy that destroys the tissue. Successful application of a super-resolution microscopy technique on intact plant cells was reported for structured illumination microscopy (SIM). This technique allowed a super-resolution time-lapse imaging of microtubule dynamics and organization in *Arabidopsis* (Komis et al., 2014) and, recently, the visualization and localization of specific domains and effector proteins at the extrahaustorial membrane of the pathogenic oomycete *Phytophthora infestans* (Bozkurt et al., 2014). In SIM applications, it is possible to achieve a lateral resolution that exceeds the classical diffraction limit by a factor of two, resulting in a possible axially resolution of 400 nm and up to 100 nm in *x*–*y* direction (Gustafsson, 2000; Schermelleh et al., 2008). This relatively high resolution can be exceeded by a factor of five in localization microscopy, which is one of the most dynamic and evolving fields of nanoscale imaging. In localization microscopy techniques such as photoactivated localization microscopy (PALM; Betzig et al., 2006) or stochastic optical reconstruction microscopy (STORM; Rust et al., 2006; Heilemann et al., 2008), a lateral resolution as high as 20 and 50 nm in the axial direction can be achieved (Bates et al., 2008). Like SIM, localization microscopy has already been successfully applied on intact plant tissue to visualize and localize cell wall polymers with a nanoscale resolution of below 50 nm (Liesche et al., 2013; Eggert et al., 2014). It turned out that both, the cellulose-specific fluorescent dye pontamine fast scarlet 4B (S4B; Anderson et al., 2010) and the callose-specific fluorescent dye aniline blue fluorochrome (ABF; Evans et al., 1984) revealed stochastic intensity fluctuations and photoblinking in stained cell walls (Eggert et al., 2014), which is a prerequisite for an application in localization microscopy. Besides a confirmation of the previously known orientation and size of the S4B-stained cellulose fibrils, new information about the interaction of cellulose and callose during pathogen attack was provided. Callose fibrils migrated into and penetrated through the preexisting cellulosic cell wall, which resulted in the formation of a three-dimensional polymer network (Eggert et al., 2014). This is a first example where localization microscopy helped to uncover previously unknown, plant immunity-related alterations and rearrangements of cell wall precisely at the site of attempted fungal penetration. Hence, localization microscopy can be used to examine localized cell wall changes induced either by stress or processes related to maintain cell wall integrity. In this regard, it would be useful to test additional fluorescent dyes that specifically label cell wall components or polymers other than cellulose and callose to receive a complete, three-dimensional overview of the cell wall and its changes in response to different types of stress. An alternative for fluorescent dyes would be the use of antibodies that are specific for different polymers or oligosaccharides of the plant cell wall. These antibodies are already known and have been tested for their specificity in intact plant tissue (Pattathil et al., 2010). For their application in localization microscopy, the primary antibodies could be either directly labeled, for example with fluorophores like CAGE552 that belongs to a class of caged rhodamines (Belov et al., 2010), or detected by an appropriate secondary antibody, which was successfully tested for the pathogen-induced callose deposition (Eggert et al., 2014). However, the use of primary and secondary antibody can decrease the maximum resolution compared to direct labeling (Ries et al., 2012; Eggert et al., 2014). In a next step, localization microscopy of cell wall polymers and components could be combined with the detection of tagged proteins within the cell wall, the apoplast, or the plasma membrane. The plasma membrane currently represents the *z*-direction limit in localization microscopy of intact, uncut plant tissue because this imaging technique is usually combined with total internal reflection microcopy resulting in a restriction of imaging to approximately 100–200 nm in *z*-direction (Cleemann et al., 1997; Martin-Fernandez et al., 2013). However, this limitation of imaging in localization microscopy would be sufficient to analyze important processes related to cell wall rearrangement as indicated in Figure 1.

The opportunities that localization microscopy already offers for nanoscale imaging of intact plant tissue, and that will likely be expended in the future, raises the question whether this new imaging tool would replace FRET due to the possibility that tagged proteins and labeled molecules could be directly imaged. At the current technical status of localization microscopy, this is not very likely because this imaging technique is limited in practice to resolutions of tens of nanometers, which is still far above the distance of FRET interactions with 1–10 nm and would not be sufficient to conclusively prove direct molecular interactions. In a future perspective, the implementation of super-resolution FRET microscopy as described by Grecco and Verveer (2011) could be a strategy to overcome limitations of both techniques and combine their superior benefits.

## Conflict of Interest Statement

The authors declare that the research was conducted in the absence of any commercial or financial relationships that could be construed as a potential conflict of interest.
